# Affective disorders—developments of ICD-11 in comparison with ICD-10

**DOI:** 10.1007/s00115-025-01877-9

**Published:** 2025-09-11

**Authors:** Martin Härter, Frank Schneider

**Affiliations:** 1 Department of Medical Psychology, University Medical Center Hamburg-Eppendorf, Hamburg, Germany; 2 Department of the History, Philosophy and Ethics of Medicine, Heinrich-Heine-University Düsseldorf, Moorenstr. 5, 40225 Düsseldorf, Germany

**Keywords:** Depressive disorder, Bipolar disorder, Cluster, Classification, ICD-11, Depressive Störung, Bipolare Störung, Cluster, Klassifikation, ICD-11

## Abstract

With the introduction of the 11th revision of the World Health Organization (WHO) “International Statistical Classification of Diseases and Related Health Problems” (ICD-11), structural and content-related adjustments were made to the diagnostic guidelines for affective disorders, which are presented in this review article. The update has resulted in some changes to the diagnostic classification of affective disorders, based on the American Diagnostic and Statistical Manual of Mental Disorders 5 (DSM-5). The ICD-11 assigns depressive symptoms to so-called clusters, the main symptoms of depressed mood and joylessness can be accompanied by cognitive, behavioral or neurovegetative symptoms. In the case of remission of depressive episodes, the ICD-11 distinguishes between partial and complete remission. A persistent depressive disorder is present if the depressive episode lasts continuously for more than 2 years. In future, bipolar disorder will be divided into type I and type II. Manic episodes can still only be coded in the context of bipolar disorders and cannot be diagnosed as an independent, separate disorder. The concept of persistent affective disorders in the ICD-10 is abandoned, dysthymia is categorized as a depressive disorder and cyclothymia as a bipolar disorder.

## Abstract (de)

Mit Einführung der 11. Revision der Internationalen statistischen Klassifikation der Krankheiten und verwandter Gesundheitsprobleme (ICD-11) der World Health Organization wurden strukturelle und inhaltliche Anpassungen der diagnostischen Richtlinien für affektive Störungen vorgenommen, die in diesem Übersichtsartikel dargestellt werden. Durch die Aktualisierung ergeben sich einige Änderungen bez. der diagnostischen Einteilung der affektiven Störungen, angelehnt an das amerikanische Diagnostic and Statistical Manual of Mental Disorders 5 (DSM-5). Die ICD-11 ordnet depressive Symptome sog. Clustern zu, die Hauptsymptome depressive Stimmung und Freudlosigkeit können von kognitiven, verhaltensbezogenen oder neurovegetativen Symptomen begleitet sein. Eine persistierende depressive Störung liegt vor, wenn die depressive Episode ununterbrochen mehr als 2 Jahre andauert. Eine bipolare Störung wird künftig in Typ I und Typ II unterschieden. Manische Episoden sind weiterhin nur kodierbar im Rahmen bipolarer Störungen und können nicht als eigenständige, separate Störung diagnostiziert werden. Das Konzept der anhaltenden affektiven Störungen in der ICD-10 wird verlassen, Dysthymie wird den depressiven Störungen und Zyklothymie den bipolaren Störungen zugeordnet.

## Background

Affective disorders are a group of disorders whose main characteristics are a change in mood, usually in phases, toward the depressive or manic pole and a change in energy. Bipolar affective disorders, in which hypomanic or manic as well as depressive or mixed episodes with more or less symptom-free intervals occur, are to be distinguished from unipolar depressive courses.

The aim of this article is to provide a concise and clear presentation of the changes in the classification of affective disorders in the transition from the International Statistical Classification of Diseases and Related Health Problems 10th Revision (ICD-10) to ICD-11. On the one hand, the taxonomic classification of depressive and bipolar disorders is presented in tabular form, and on the other hand, specific information on the diagnosis of depressive disorders is given. For more detailed information, reference is made to the corresponding classification systems of the comprehensive disease categories including their specifics, available from the Federal Institute for Drugs and Medical Devices (BfArM)^1^^,^^2^.

### Changes in the taxonomy of affective disorders in the transition from ICD-10 to ICD-11

The transition from ICD-10 to ICD-11 resulted in the following structural changes (see also [1–7]):

In addition to the classification into *unipolar and bipolar courses*, episodic affective disorders, i.e., *manic* (Chaps. F30–31) or *depressive episodes* (Chaps. F.32–33), were distinguished from *chronic persistent affective disorders* (dysthymia, cyclothymia) in ICD-10 (Chap. F.34). In addition, there were the residual categories “Other affective disorders” (Chap. F.38) and “Affective disorder not otherwise specified” (Chap. F.39).

The diagnosis of affective disorders remains *categorical* in principle in ICD-11, but the extent of operationalization (i.e., counting symptoms) has been reduced in favor of a clinical assessment with the central criteria of symptom severity and functional impairment. The concept of persistent affective disorders of ICD-10 is abandoned in ICD-11; *dysthymia* is assigned to *depressive disorders* and *cyclothymia* to *bipolar disorders*.

**Fig. 1:**
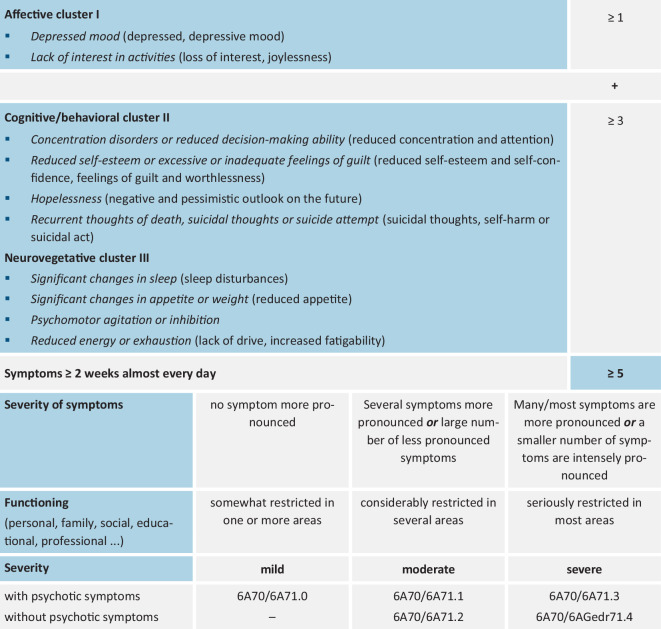
Diagnostic guidelines (ICD-11): depressive disorder with single episode or recurrent depressive disorder (symptoms/criteria of ICD-10 in parentheses)

In ICD-11, the *differentiation of depressive symptoms* into three symptom clusters, a dimensional assessment of severity, and a greater degree of freedom for clinical professionals in the diagnosis of affective disorders are realized (see Fig. 1). In addition, the additional coding “with psychotic symptoms” also exists for moderate depressive episodes. In the case of currently remitted depressive episodes, ICD-11 also distinguishes between *partial *and *complete remission*.

“*Premenstrual dysphoric disorder*” and “*Mixed depressive disorder and anxiety disorder*” are included as new categories.

After the first manifestation of a depressive episode, the majority of patients (55–65%) experience several depressive phases over the course of their lives (*recurrent depressive disorder*). The risk of relapse increases with each subsequent depressive episode. In addition, as the number of episodes increases and with increasing age, the intervals between depressive phases and recurrent depressive disorder become shorter (see taxonomy of recurrent depression; ICD-10 vs. CD-11, Table 1).

**Table 1:** Taxonomy of depressive disorders in ICD-10 and ICD-11

ICD-10	ICD-11 (draft version)
*Affective disorders (F30–31)*	*Affective disorders (6A60.X–6A73.X)*
*F32.—Depressive episode*	*6A70 Single-episode depressive disorder*
F32.0 Mild depressive episode	6A70.0 Single-episode depressive disorder, mild
F32.1 Moderate depressive episode	6A70.1 Single-episode depressive disorder, moderate, without psychotic symptoms 6A70.2 Single-episode depressive disorder, moderate, with psychotic symptoms
F32.2 Major depressive episode without psychotic symptoms	6A70.3 Single-episode depressive disorder, severe, without psychotic symptoms
F32.3 Depressive disorder, current major episode with psychotic symptoms	6A70.4 Single-episode depressive disorder, severe, with psychotic symptoms
F32.4 Depressive disorder, currently remitted	6A70.6 Depressive disorder with single episode, currently in partial remission 6A70.7 Depressive disorder with single episode, currently in full remission
F32.8 Other depressive episodes Atypical depression	6A8Y Other specified affective disorders
F32.9 Depressive episode, unspecified	6A8Z Affective disorders, unspecified
*F33.—Recurrent depression*	6A71 Recurrent depressive disorder
F33.0 Recurrent depressive disorder, currently mild episode	6A71.0 Recurrent depressive disorder, current mild episode
F33.1 Recurrent depressive disorder, current moderate episode	6A71.1 Recurrent depressive disorder, current moderate episode, without psychotic symptoms 6A71.2 Recurrent depressive disorder, current moderate episode, with psychotic symptoms
F33.3 Recurrent depressive disorder, current major episode without psychotic symptoms	6A71.3 Recurrent depressive disorder, current severe episode, without psychotic symptoms
F33.3 Recurrent depressive disorder, current major episode with psychotic symptoms	6A71.4 Recurrent depressive disorder, current major episode, with psychotic symptoms
F33.4 Recurrent depressive disorder, currently remitted	6A71.6 Recurrent depressive disorder, currently in partial remission 6A71.7 Recurrent depressive disorder, currently in full remission
F33.8 Other recurrent depressive disorder	6A71.Y Other specified recurrent depressive disorder
F33.9 Recurrent depressive disorder, unspecified	6A71.Z Recurrent depressive disorder, unspecified
*F34.—Persistent affective disorders*	–
F34.1 Dysthymia	6A72 Dysthymic disorder
F34.8 Other persistent affective disorders	–
F34.8 Persistent affective disorder, unspecified	–
*New categories*	–
F41.2 Anxiety and depressive disorder, mixed	6A73 Mixed depressive and anxiety disorder
–	GA34.41 Premenstrual dysphoric disorder

*ICD *International Statistical Classification of Diseases and Related Health Problems

In future, *bipolar disorder* will be divided into *type I* and *type II*. Manic episodes can still only be coded in the context of bipolar disorders and cannot be diagnosed as an independent, separate disorder (see Table 2).

**Table 2:** Taxonomy of bipolar disorders in ICD-10 and ICD-11

ICD-10	ICD-11 (draft version)
*Affective disorders (F30–39)*	*Affective disorders (6A60.X–6A61.X)*
*F30.—Manic episode*	*6A60 Bipolar disorder type I*
F30.0 Hypomania	6A60.2 (or 6A61.2) Bipolar disorder type I (or type II), current hypomanic episode
F30.1 Mania without psychotic symptoms	6A60.0 Bipolar disorder type I, current manic episode, without psychotic symptoms
F30.2 Mania with psychotic symptoms	6A60.1 Bipolar disorder type I, current manic episode, with psychotic symptoms
F30.8 Other manic episodes	6A8Y Other specified affective disorders
F30.9 Manic episode, unspecified	6A8Z Affective disorders, unspecified
*F31.—Bipolar affective disorder*	*6A60 Bipolar disorder type I*
F31.0 Bipolar affective disorder, current hypomanic episode	6A60.2 (or 6A61.2) Bipolar disorder type I (or type II), current hypomanic episode
F31.1 Bipolar affective disorder, current manic episode without psychotic symptoms	6A60.0 Bipolar disorder type I, current manic episode, without psychotic symptom
F31.2 Bipolar affective disorder, current manic episode with psychotic symptoms	6A60.1 Bipolar disorder type I, current manic episode, with psychotic symptoms
F31.3 Bipolar affective disorder, current mild or moderate depressive episode	6A60.3 Bipolar disorder type I, current mild depressive episode 6A60.4 Bipolar disorder type I, current moderate depressive episode, without psychotic symptoms
F31.4 Bipolar affective disorder, current major depressive episode without psychotic symptoms	6A60.6 Bipolar disorder type I, current major depressive episode, without psychotic symptoms
F31.5 Bipolar affective disorder, current major depressive episode with psychotic symptoms	6A60.7 Bipolar disorder type I, current major depressive episode, with psychotic symptoms
F31.6 Bipolar affective disorder, current mixed episode	6A60.9 Bipolar disorder type I, current mixed episode, without psychotic symptoms 6A60.A Bipolar disorder type I, current mixed episode, with psychotic symptoms
F31.7 Bipolar affective disorder, currently remitted	6A60.F Bipolar disorder type I, currently in full remission
F31.8 Other bipolar affective disorder	6A60.Y Other specified bipolar disorder type I
F31.9 Bipolar affective disorder, unspecified	6A60.Z Bipolar disorder type I, unspecified
*–*	*6A61 Bipolar disorder type II*
–	6A61.0 Bipolar disorder type II, current hypomanic episode
–	6A61.1 Bipolar disorder type II, current mild depressive episode
–	6A61.2 Bipolar disorder type II, current moderate depressive episode, without psychotic symptoms
–	etc.
*F34.—Persistent affective disorders*	–
F34.0 Cyclothymia	6A62 Cyclothymic disorder
F34.8 Other persistent affective disorders	–
F34.9 Persistent affective disorder, unspecified	–

*ICD *International Statistical Classification of Diseases and Related Health Problems, *etc.* Continue coding as for bipolar disorder type I to 6A61.Z bipolar disorder type II, unspecified

To capture inter-individual differences, depressive or manic episodes can be additionally coded with so-called *qualifiers* (6A80*) (analogous to the “specifiers” of the *Diagnostic and Statistical Manual of Mental Disorders, Fifth Edition* [DSM-5]), e.g., with regard to accompanying anxiety syndromes, a chronic course, a seasonal pattern, or in relation to the peripartum period [4]. In the ICD-11, it is possible to code further clinical manifestations in addition to the affective core diagnosis of “depression” or “bipolar disorder”, e.g., “with marked anxiety symptoms” (6A80.0); “with panic attacks” (6A80.1); “with persistent symptoms,” i.e., at least 2 years (= possibility of coding chronic depression that goes beyond dysthymia; 6A80.2); “with melancholia” (6A80.3, which was coded as “somatic syndrome” in ICD-10); “with seasonal onset” (6A80.4); or “with rapid cycling” (6A80.5).

## Diagnosis of depressive episodes

To diagnose a depressive episode, *at least five symptoms* must be present (four were sufficient in ICD-10), *at least one of which must be from the affective cluster*. The classification of episode severity (mild, moderate, severe) is—unlike in the ICD-10—not based on the sum of the symptoms, but also takes into account their *intensity* and the *degree of functional impairment* in addition to the number of symptoms. The grouping of depression symptoms is intended to illustrate the wide clinical range of symptoms and increases clarity. Cluster I contains the two so-called entry-level symptoms. This no longer includes the less specific symptom “lack of energy, increased fatigability,” which now belongs to Cluster III as “reduced energy or exhaustion.”

## Concluding remarks

It is to be hoped that the new ICD-11 will be met with widespread acceptance once it has been introduced into healthcare in Germany. Although the coding will be more differentiated and, if necessary, more detailed, this also corresponds to the general efforts to achieve even greater personalization with regard to the treatment strategies to be used. The greater emphasis on the dimensionality of disorders, e.g., with regard to functional impairment and severity, can contribute to the fact that, in addition to the clinical reduction of symptoms, the improvement of functional limitations and quality of life in particular will become more important as therapeutic goals.

## Practical conclusion

- In the case of transient depressive moods, which are very common in the general population, it makes sense to differentiate them from depressive disorders by assessing the severity of symptoms and functional limitations.
- The grouping or clustering of depressive symptoms is useful due to the clinical breadth of the symptomatology and it increases clarity.
- The addition of persistent or chronic depression was overdue and was already frequently used in everyday clinical practice.
- The addition of the “qualifiers” to the classification makes clinical sense, as numerous symptoms such as anxiety, panic, persistence, seasonal pattern, or melancholy are common and useful for treatment planning.
- The abandonment of the coding of individual manic episodes is logical, as the separation was not well documented in the context of family, therapy, and follow-up studies.
- The independent coding of bipolar disorder type 2 is welcome, since study findings support this view and the prognosis and response to treatment are usually more favorable.
- The resolution of the persistent affective disorders and the classification in the respective chapters of depressive and bipolar disorders is particularly helpful because of the similar treatment strategies; in addition, clinical courses with transitions are often found in these subcategories.

## Footnotes

1 BfArM—ICD-10-GM Version 2024.

2 BfArM—ICD-11 in German—Draft Version.

## Declarations

For this article no studies with human participants or animals were performed by any of the authors. All studies mentioned were in accordance with the ethical standards indicated in each case.

The supplement containing this article is not sponsored by industry.
